# Susceptibility to Clotrimazole of *Candida* spp. Isolated from the Genitourinary System—A Single Center Study

**DOI:** 10.3390/pathogens10091142

**Published:** 2021-09-04

**Authors:** Magdalena Frej-Mądrzak, Sabina Golec, Katarzyna Włodarczyk, Irena Choroszy-Król, Urszula Nawrot

**Affiliations:** 1 Department of Basic Sciences, Faculty of Health Sciences, Wroclaw Medical University, 50-367 Wroclaw, Poland; irena.choroszy-krol@umed.wroc.pl; 2 Department of Pharmaceutical Microbiology and Parasitology, Faculty of Pharmacy, Wroclaw Medical University, 50-367 Wroclaw, Poland; sab.golec@gmail.com (S.G.); katarzyna.wlodarczyk@umed.wroc.pl (K.W.); urszula.nawrot@umed.wroc.pl (U.N.)

**Keywords:** *Candida*, drug susceptibility, clotrimazole, vulvovaginal

## Abstract

The aim of this study was to determine the susceptibility to clotrimazole of 125 isolates of *Candida* spp. originated from the genitourinary system of hospitalized patients as well as outpatients, tested in the mycological laboratory of Wroclaw Medical University in the years 1999–2018. The minimal inhibitory concentrations of clotrimazole and fluconazole were determined with the use of the microdilution method according to EUCAST, and the MFC was determined by subsequent subculture on Sabouraud agar. For the tested population of *Candida* yeasts, the MIC values of clotrimazole ranged from 0.008 to 8 mg/L, and MIC90 was 1 mg/L, whereas MIC50 was 0.008 mg/L. The minimal fungicidal concentration ranged between 1 and >8 mg/L. The great majority of the isolates (88%; 110/125) displayed MIC < 1 mg/L and were classified as WT (wild-type), whereas MIC ≥ 1 mg/L was determined for 2/61 (3.2%) isolates of *C. albicans*, 9/38 (23.6%) of *C. glabrata*, 1/2 of *C. tropicalis*, and 3/3 of *C. guilliermondii.* Six isolates (four of *C. glabrata* and two of *C. albicans*), defined as non-WT for clotrimazole, were classified as resistant to fluconazole, according to CBP from EUCAST. The isolates with elevated MIC to clotrimazole originated mostly from patients of the pediatric hematology unit, and their proportion in this population amounted to 17.8% (13 out of 73 isolates). In contrast, among strains from ambulatory patients, the highest observed MIC value was 1 mg/L (1 out of 37 isolates; 2.7%). The data obtained correlate well with those of most published studies on the in vitro susceptibility of *Candida* spp. to clotrimazole, which is usually very high. However, the existence of reports regarding the growing prevalence of resistant isolates has also to be noted. These results support the need for routinely checking the susceptibility of *Candida* clinical isolates to this imidazole derivative.

## 1. Introduction

*Candida* spp. are opportunistic pathogens and an element of the natural microbiota of the human skin and the mucosa of the gastrointestinal and genitourinary systems. At the same time, they are one of the most frequently isolated fungal pathogens involved in superficial as well as deep-seated mycoses. Candidal infections are frequently localized in the mucous membranes of the genitourinary system, and infections occur both in women and men; however, vulvovaginal mycoses are more common. The main pathogen of vulvovaginal candidiasis (VVC) is *Candida albicans*, which is responsible for up to 85% of cases of this disease. This condition often arises in patients using hormonal contraceptives, antibiotic therapies, vaginal irrigations, or other procedures leading to disturbance of the microbiota of the vagina and vulva. About 15% of women with VVC develop a recurrent form of the disease (rVVC), which has been defined as manifesting in at least three (or four) episodes per year [1,2,3].

In addition to *C. albicans*, other species of this genus (so-called *Candida* non-*albicans*), most commonly *Candida glabrata*, can cause episodic and recurrent VVC. *Candida* non-*albicans* dominate especially in immunosuppressed people, including those with congenital immunodeficiency who, due to the risk of infections, often receive antifungal prophylaxis. In such patients, chronic mucocutaneous candidiasis may be encountered, where the urogenital system may be one of the locations of multifocal mycosis. Candidiasis of the mucous membranes is therefore a clinical problem on a global scale, and due to its high frequency, it also represents an important economic issue. Azoles and polyenes are primarily used in antifungal therapy, but the clinical effectiveness of the therapy is not always satisfactory, as evidenced by reinfections and chronic infections. Clotrimazole is one of the topical drugs most commonly used for candidiasis, although its uncontrolled and/or incorrect use, e.g., prolonged or repeated short-term drug exposure, may contribute to the selection of resistant strains. Whilst there is also a risk of cross-resistance to other azole preparations, currently, there are insufficient clinical data on the development of resistance of the genus *Candida* to clotrimazole, and susceptibility to this drug is not routinely tested. The aim of this study was to determine the susceptibility to clotrimazole of a collection of 125 isolates of *Candida* spp. originated from the genitourinary system of hospitalized as well as outpatients, tested in the mycological laboratory of Wroclaw Medical University in the years 1999–2018. The results obtained were compared with data from the literature in terms of the assessment of *Candida* susceptibility to clotrimazole, the methods used, and the interpretative criteria.

## 2. Results

### Susceptibility to Clotrimazole and Fluconazole

**Figure 1:**
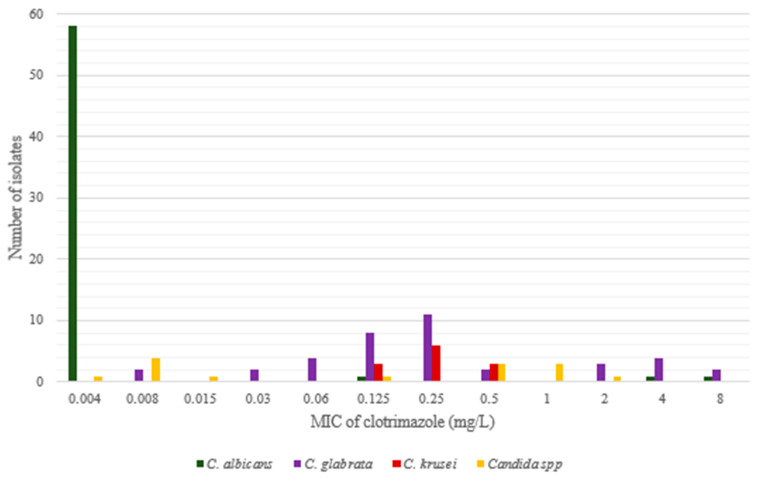
Clotrimazole MIC values for yeasts tested in this study.

Detailed results of the minimal inhibitory concentration of clotrimazole obtained for each of the 125 tested isolates as well as the MIC of fluconazole determined for 15 isolates are presented in Table S1 (Supplementary). The quantitative distribution of clotrimazole MIC values for particular species is shown in Table 1 and Figure 1. 

The study indicated that most of *C. albicans* isolates (58/61, 95.08%) exhibited clotrimazole MICs below 0.008 mg/L; MIC for one isolate was 0.125 mg/L, and that for two other isolates was 4 and 8 mg/L. The last-mentioned isolates also showed elevated MIC to fluconazole and, according to EUCAST breakpoints [4], were classified as susceptible, increased exposure (one isolate), or resistant (two isolates) to this drug (Table 2 and Table 3).

**Table 1:** MIC values of clotrimazole in the tested population of *Candida* species and comparison with results published by other authors with the use of the CLSI method.

Species (Number of Isolates)	Number of Isolates at MIC [mg/L]												MIC50	MIC90
	<0.008	0.008	0.015	0.03	0.06	0.125	0.25	0.5	1	2	4	8		
*C. albicans* (61)	58					1					1	1	<0.008	<0.008
*C. albicans* (420); Richter et al. [5]		9	92	252	65	2							0.03	0.06
*C. glabrata* (38)		2		2	4	8	11	2		3	4	2	0.25	4
*C. glabrata* (250) Richter et al. [5], Costa et al. [6];				1	7	15	21	21	53	45	36	21		
*C. krusei* (12)						3	6	3					0.25	0.5
*C. krusei* (12) Richter et al. [5]						2	5	4	1				0.25	0.5
*C. inconspicua* (3)						1		2						
*C. guilliermondii* (3)									2	1				
*C. parapsilosis* (2)		1	1											
*C. tropicalis* (2)		1							1					
*C. dubliniensis* (1)	1													
*C. lusitaniae* (1)		1												
*C. pararugosa* (1)								1						
*C. melibiosica* (1)		1												
non-*albicans.* (64)	1	6	1	2	4	12	17	8	3	4	4	2	0.25	2
Total (125)	59	6	1	2	4	13	17	8	3	4	5	3	0.008	1

In the population of *C. glabrata*, most isolates showed clotrimazole MIC between 0.008 and 0.5 mg/L; nevertheless 9 out of 38 (23.6%) isolates displayed MIC above 1 mg/L (2–8 mg/L). Five isolates of this subgroup were classified as resistant to fluconazole (Table 2). The clotrimazole MIC values for *Candida krusei* were subdivided in a narrow range from 0.125 to 0.5, and those for *Candida parapsilosis* from 0.015 to 0.03; MIC for *Candida tropicalis* was 0.008 for one strain and 1 mg/L for the second one. For three isolates of *Candida guilliermondii*, the MIC of clotrimazole was 1 mg/L (two strains) and 2 mg/L (one isolate), whereas the MIC of fluconazole for these isolates was 4 mg/L (wild-type phenotype according to EUCAST). Among *Candida* non-*albicans*, the lowest MIC values were found for *Candida dubliniensis* (<0.008 mg/L) and for representatives of *Candida lusitaniae* and *Candida melibiosica* (0.008 mg/L). The clotrimazole MIC50 (the lowest concentration of clotrimazole inducing growth inhibition of 50% in the tested population of isolates) for the whole population of tested *Candida* was 0.008 mg/L, while the MIC90 (the lowest concentration of clotrimazole inducing growth inhibition of 90% in the tested population of isolates) was 1 mg/L. The minimal fungicidal concentration ranged between 1 and >8 mg/L. The ratio of MFC/MIC ranged from 1 (for one isolate of *C. glabrata*) to >500 for *C. albicans* (Table S1). The clotrimazole MICs for the reference strains were as follows: *C. glabrata* ATCC 90030, 0.125 mg/L, *C. albicans* ATCC 90028, 0.008 mg/L, *C. albicans* ATCC 10231, 0.03 mg/L, and *C. krusei* ATCC 6258, 0.125 mg/L.

## 3. Discussion 

Clotrimazole, an imidazole derivative introduced to the market about 45 years ago, is still one of the most commonly used topical antimycotics in the treatment of mucocutaneous candidiasis. A long-term treatment in cases of chronic or recurrent infections, e.g., rVVC, as well as an improper dosage facilitated by the universal access to this medicine (usually available on medical prescription), pose a risk of the development of strains with acquired resistance to azoles. Surprisingly, cases of such resistance are reported relatively seldom, which may also be due to the fact that, usually, susceptibility to this drug is not checked by routine microbiological testing. For this reason, there is no comprehensive knowledge concerning the presence of clotrimazole resistance and the risk of its emergence during treatment [21]. 

In this study, we estimated the susceptibility to clotrimazole of 125 strains of *Candida* spp. isolated from the mucous membranes of the genitourinary system. The isolates represented a heterogeneous group in terms of origin. The samples were isolated from adults (54/125 isolates), from symptomatic infections, while the controls (73 isolates) were obtained during screening from usually asymptomatic children hospitalized in the hematology clinic. Overall, in the studied population of strains, 48.8% (61/125) of the isolates were *C. albicans*, while the next most frequent species were *C. glabrata* 30.4% (38/125) and *C. krusei* 9.6% (12/125). All other species of *Candida* accounted for 11%. The proportion of *Candida* spp. (*C*. non-*albicans*) among the isolates from the children was higher (48/73; 65%) than among the isolates from the adults (27%), which may be related to the use of antifungal prophylaxis effective against *C. albicans* (e.g., fluconazole), which is a standard procedure in hematology. The analysis of the susceptibility to clotrimazole indicated that 88% (110/125) of *Candida* spp. isolates showed MIC values lower than 1 mg/L. All but two isolates with a MIC ≥ 1 mg/L originated from the patients of the pediatric hematology unit; thus, their proportion in this population amounted to 17.8% (13 out of 73 isolates). In contrast, among strains from ambulatory patients, the highest observed MIC value was 1 mg/L (1 out of 37 isolates; 2.7%). The MIC of 0.5 mg/L (value used as a cut-off in some studies) was determined for eight isolates of *Candida* non-albicans, and the overall proportion of isolates with a MIC ≥ 0.5 mg/L was 18.4% (23/125).

The frequency of clotrimazole resistance reported by other authors differs from study to study and could be related not only to the epidemiological resources but also to the method of susceptibility testing. In the present study, we applied the reference microdilution method, recommended by EUCAST. A great difficulty related to this method is the interpretation of the MIC results, which is based on clinical breakpoint values, the determination of which requires not only extensive microbiological examination but also clinical analysis. Currently, neither EUCAST nor CLSI have published data regarding both clinical and epidemiological MIC breakpoints for clotrimazole. It is well known that the susceptibility to antimicrobials and MIC values are species-specific. *C. albicans* and closely related species (*C. africana, C. dubliniensis*) are regarded as very sensitive to azoles, including clotrimazole. This rule was confirmed by several studies (including this article), in which MIC90 for these species ranged between 0.03 and 0.5 mg/L (Table 3) [6,7,8,9,10,11,12,13,15]. 

Pelletier et al., in their study performed 20 years ago on 87 *C. albicans* isolates, suggested a MIC ≥ 0.5 mg/L as a tentative breakpoint indicating resistance [12]. In the above-mentioned study, 17% (15/87) of the tested isolates showed a MIC ≥ 0.5 mg/L and were regarded as resistant. The authors found that a MIC of clotrimazole ≥ 0.5 mg/L correlated with a significant risk of cross-resistance to other azoles such as fluconazole and itraconazole. A few years later, Richter et al. [5] published a study in which they examined the susceptibility to clotrimazole and other azoles of almost 600 clinical yeast isolates obtained from the genital tract and adopted a MIC of 1 mg/L as the cut-off for resistance to clotrimazole for all *Candida* species included in the study. However, none of the 420 strains of *C. albicans* tested by Richter et al. showed a MIC higher than 0.125 mg/L. By contrast, in the study of Nelson et al. [14], the percentage of clotrimazole-resistant *C. albicans* was 36.6%. 

A very interesting study was described by Marchaim et al. [22], who analyzed cases of recurrent vaginitis due to fluconazole-resistant *Candida albicans*. Patients were exposed to a long-term treatment with fluconazole, and most of them received a low dose of fluconazole weekly during a 12 month period before the isolation of a resistant strain. The isolates with an elevated MIC of clotrimazole (≥ 0.5 mg/L) were resistant to fluconazole, with a MIC of 4–128 mg/L. Similarly, in our study, both detected isolates of *Candida albicans* with clotrimazole MIC > 0.5 mg/L were cross-resistant to fluconazole. As mentioned above, resistant strains were obtained from children from the hematological department, who, similarly to patients with rVVC described by Marchaim et al., received antifungal therapy. Cross resistance between clotrimazole and other azoles was described by other authors, e.g., Khadka et al. [23], who used a disc diffusion method recommended by the Clinical and Laboratory Standards Institute (CLSI, M44-A). Among 56 *C. albicans* isolates, 8 (14.3%) were classified as SDD (susceptible, dose-dependently), and 4 (7.2%) as resistant to clotrimazole. The strains reported to be resistant to clotrimazole showed cross resistance to other azoles, including fluconazole [23]. 

*C. glabrata* isolates with known lower susceptibility to azoles (especially, fluconazole) represent the second most frequent cause of candidosis of the genitourinary tract. The isolates of *C. glabrata* included in this study showed a wide range of MIC values for clotrimazole (between 0.015 mg/L and 8 mg/L). Most isolates (29/38; 76.3%) displayed a MIC ≤ 0.5 mg/L, whereas 23% (9/38) of them showed MIC > 0.5 mg/L and were regarded as non-wild-type (non-WT). The MIC50 of the entire pool of the tested *C. glabrata* strains was 0.25 mg/L, while the MIC90 was 4 mg/L. The values of MIC90 of *C. glabrata* obtained by many other authors ranged between 1 and 4 mg/L (Table 3) [5,7,9,10,11,13,14,16,19]. An exception are the results obtained by Costa et al. [6], who analyzed *C. glabrata* isolated from different clinical samples of hospitalized patients, previously exposed to antifungal therapy. The authors found 64% of the tested isolates resistant to clotrimazole (MIC90 equal to 8 mg/L) and, simultaneously, 15% of them resistant to fluconazole (MIC ≥ 32 mg/L). Similarly, in the present study, five out of nine *C. glabrata* isolates with an elevated MIC of clotrimazole proved to be resistant to fluconazole, with an MIC ≥ 32 mg/L (Table 2). This indicates that these isolates harbor a resistance mechanism resulting in a simultaneous loss of susceptibility to both clotrimazole and fluconazole. The strains with pan-azole resistance could be selected during oral therapy with fluconazole as well as during topical treatment with over-the-counter azoles, e.g., clotrimazole. Such thesis supports the study by Cross et al., who observed the spontaneous development of resistant *C. glabrata* mutants under the selective pressure of clotrimazole [24]. Costa et al. [6] demonstrated that the resistance of *C. glabrata* to clotrimazole correlated with increased expression of genes encoding DHA membrane transporters (Drug: H + Antiporters): CgAqr1, CgQdr2, CgTpo1-1, and CgTpo3, a type of efflux pump related to the ABC pump family (ATP Binding Cassette). This was evidenced by a decrease in resistance of the tested strains to clotrimazole after deletion of the gene coding for CgTpo3, which resulted in increased accumulation of this drug in fungal cells. The results of this study suggest a role of the CgTpo3 transporter in the development of the resistance mechanism to clotrimazole and other azole drugs (including fluconazole) [6]. 

*C. krusei*, a relatively rare pathogen of the genital tract, is regarded as being naturally resistant to fluconazole and frequently less susceptible to other azoles. Isolates of *C. krusei* tested in the present study showed low MIC values for clotrimazole (0.125–0.5 mg/L) and belonged to the WT population, despite the fact that 50% of them were isolated from a population at risk (children from the hematology department). Similar results were obtained in studies by Richter et al. [5] and Singh et al. [20]. They obtained MIC values of clotrimazole that did not exceed 1 mg/L. Singh et al. [20] found clotrimazole to be the most potent azole derivative against *C. krusei* isolated from chronic vulvovaginal candidiasis. However, clotrimazole-resistant isolates of *C. krusei* were also reported in the literature (Nelson et al. [14]). 

The percentage of genitourinary tract infections caused by *C. parapsilosis* and *C. tropicalis* is relatively low and oscillates around 6% [25]. In our study, each of these species was represented by two isolates, one isolate of *C. tropicalis* was non-WT with a MIC of 1 mg/L, and the balance of the isolates were susceptible. The vaginal isolates of *C. parapsilosis* and *C. tropicalis* studied by other authors had usually a WT phenotype and displayed a low MIC of clotrimazole [5]. These species, similar to *C. albicans*, usually showed good susceptibility to triazoles, especially to fluconazole. An extraordinary result was reported recently by Kiakojuri et al., 2021, who tested isolates from patients with otomycosis and found that all of the *C. parapsilosis* isolates and most of the closely related *Candida orthopsilosis* ones were resistant to clotrimazole (MIC90 16 mg/L) as well fluconazole (MIC90 64 mg/L) [17]. 

In the present study, we also tested some of the less frequently encountered *Candida* species (*C. inconspicua*, *C. lusitaniae*, *C. pararugosa*, *C. melibiosica*), and most of them were susceptible to clotrimazole (MIC < 0.5 mg/L). The exception was strains of *C. guilliermondii* which showed clotrimazole MIC values of 1 or 2 mg/L and fluconazole MIC of 4 mg/L (phenotype WT in relation to fluconazole; ECOFF 16 mg/L) [4]. 

The data regarding the susceptibility of clinical isolates of *Candida* to clotrimazole, published in this study as well as in the current medical literature, indicated that the proportion of non-susceptible strains is rather low, especially in isolates obtained from the general population. An alarmingly high representation of resistant isolates has been reported mostly in high-risk populations exposed to prolonged azole (usually triazole) treatment. Evaluation and comparison of the susceptibility data of particular *Candida* species are difficult. Although clotrimazole has been used for a long time, a reliable method of interpretation of the MIC data (clinical breakpoints and epidemiological cut-off) has not yet been established. Analysis of published MIC data for *C. albicans* indicates that the tentative breakpoint proposed by Pelletier et al. [12] could be accepted for this species. On the other hand, the distribution of MIC values for *C. glabrata* indicates that its ECOFF is higher than that of *C. albicans* and a value of at least 1–2 mg/L is reasonable. The therapeutic effectiveness of antifungal therapy is not always well correlated with the susceptibility data. It especially concerns a topical treatment, when the distribution of the concentration of the compound may greatly exceed the MIC values, even for strains belonging to the non-WT population. The low water solubility, as well as the increase in the MIC value in an acidic environment (pH 4) observed for clotrimazole, may be a reason for a lower-than-expected antifungal effect [26,27]. This may be connected with the fact that the action of azole derivatives, including clotrimazole, against *Candida* species is usually fungistatic. The lack of eradication of the microorganism could result in the development of persistent infections or reinfections in the future. Among the factors influencing the efficacy of an antifungal therapy we can list patient-related errors. An improper application of the drug or its irregular use may be a secondary cause of an unsatisfactory clinical response. To improve topical treatments, new drug formulations are under development, e.g., combinations with chitosan [26], intravaginal rings [28], clotrimazole-loaded polymeric micelles with hyaluronic acid [29], and many others. 

In order to determine the effectiveness of the clotrimazole therapy as well as to develop reliable diagnostic standards of susceptibility testing, it is necessary to conduct simultaneous clinical and microbiological tests.

## 4. Materials and Methods

### 4.1. Strains

The study was performed on 125 *Candida* spp. isolates preserved in the laboratory collection of the Department of Pharmaceutical Microbiology and Parasitology of the Wroclaw Medical University. Of these, 14 isolates were incorporated from the collection of the Department of Basic Sciences, Faculty of Health Sciences of the Wroclaw Medical University. The isolates were derived during routine microbiological examinations in the years 1999–2018 and were preserved frozen at −71 °C in TSB medium supplemented with 15% glycerol. The strains originated from the following clinical materials: vaginal smears (67), swabs from the cervix (19), swabs from under the foreskin (31), and swabs from the skin or wounds (8). Most strains (88) were isolated from hospitalized patients, including those in the adult and pediatric hematology unit (74 strains), intensive care unit (7), gynecology (5), and surgery, (2) and the remaining 37 isolates were from ambulatory patients. The strains tested were identified as follows: *Candida albicans* (61), *Candida glabrata* (38), *Candida krusei* (12), *Candida guillermondii* (3), *Candida inconspicua* (3), *Candida parapsilosis* (2), *Candida tropicalis* (2), and one each of *Candida dubliniensis*, *Candida lusitaniae*, *Candida pararugosa*, and *Candida melibiosica*. Details of the origin of the strains are provided in Table S1 (Supplement). Apart from the clinical isolates, the following reference strains from the American Type Culture Collection were investigated: *Candida albicans* ATCC 90028, *Candida albicans* ATCC 10231, and *Candida glabrata* ATCC 90030.

### 4.2. Culture and Re-Identification of the Strains

For cultivation, the strains stored at −70 °C were thawed and streaked on Sabouraud Dextrose Chloramphenicol Agar (BioMaxima, Poland) medium, then incubated at 37 °C for 24–48 h. Colony morphology was assessed for culture purity, and single colonies were screened with chromogenic CHROMagar™ Candida medium (BioMaxima, Poland), which enables the differentiation of *C. albicans, C. krusei, C. tropicalis*, and *Candida* spp., as well as with Chromogenic Candida Plus Lab-Agar™, (BioMaxima, Poland), which additionally allowed the detection of the species *Candida auris*. The isolates classified as *Candida* spp. were subsequently investigated on the basis of their morphology in microculture on rice agar medium as well as of their biochemical profile with BD Phoenix™ Yeast ID tests and the Phoenix™ 100 (Becton Dickinson) system, in accordance with the manufacturer’s instructions. 

### 4.3. Determination of Susceptibility to Antifungals/Clotrimazole

Susceptibility to clotrimazole (Hasco Lek, Poland) and fluconazole (Sigma-Aldrich) was tested with the use of the microdilution method according to EUCAST E.Def 7.3.2 [30]. Briefly, the investigation was carried out in liquid RPMI 1640 medium supplemented with 2% glucose and buffered with MOPS (3-(N-morpholino) propanesulfonic acid) (Sigma Aldrich); 96% ethanol was used to dissolve clotrimazole, and sterile distilled water to dissolve fluconazole. 

A stock solution of clotrimazole of 1600 mg/L in 96% ethanol was used to prepare a series of 2-fold ethanol solutions at concentrations ranging from 1.562 mg/L to 800 mg/L. Subsequently, they were diluted again in double-strength RPMI 16,402×, to obtain clotrimazole concentrations ranging from 0.0156 mg/L to 16 mg/L. The prepared solutions were dispensed in a volume of 50 µL into the appropriate wells of 96-well microplates. In the case of fluconazole, the drug concentrations in the microtiter plates ranged from 0.25 mg/L to 128 mg/L. The microplates were preserved frozen at −76 °C until used. Before drug susceptibility testing, the investigated strains were cultured at 37 °C for 24 h on Sabouraud dextrose agar medium. Fungal cells were suspended in distilled water to obtain a density of 0.5 McFarland (1–5 × 10^6^ CFU/mL) and thereafter diluted 1 in 10 to obtain 1–5 × 10^5^ CFU/mL. The prepared inoculum of each strain was applied in a volume of 50 µL to the wells with antimycotics as well as to growth control (RPMI 16402× without antimycotics). The final density of the fungal suspension (applied to the microtiter plate) was in the range 0.5–2.5 × 10^5^ CFU/mL, and the final clotrimazole concentrations were 0.006–8 mg/L, whereas those of fluconazole were 0.125–64 mg/L. All inoculation activities were performed under sterile conditions in a laminar-flow cabinet. Inoculated microplates were incubated for 24 h at 37 °C. The absorbance of the samples in each well was then measured by a Multiscan Go spectrophotometer at a wavelength of 530 nm. The MIC value was determined as the lowest concentration of the antimycotic expressed in mg/L at which the absorbance value was at least 50% lower than that of the control strain in the same medium without the antibiotic. The interpretation of the results was based on the clinical breakpoints published by EUCAST (fluconazole) [17] and the publication of Richter et al. and Pelletier et al. (clotrimazole) [7,12].

### 4.4. Determination of MFC, the Minimal Fungicidal Concentration of Clotrimazole 

After microplate incubation and determination of the MIC values for 22 randomly selected strains, the minimum fungicidal concentration (MFC) of clotrimazole was determined. For this purpose, an aliquot of 20 µL of the culture medium was taken from a well of the microplate, where no growth (no turbidity) was observed and plated on Sabouraud dextrose agar plates. Subsequently, these media were incubated at 37 ° C for 24–48 h. The lowest concentration at which ≤1 colony growth was observed was considered the MFC.

## 5. Conclusions

*Candida albicans* was highly sensitive to clotrimazole. Strains with a non-WT phenotype which were detected in only two patients of the oncology department. The MICs of clotrimazole for *Candida* non-*albicans* strains were higher than those of for *Candida albicans*, and the non-WT strains most often belonged to the species *Candida glabrata*. Strains with the non-WT phenotype with respect to clotrimazole showed different sensitivity to fluconazole (cross resistance to fluconazole and clotrimazole or resistance to clotrimazole only). This suggests the existence of various resistance mechanisms and indicates the need to measure drug susceptibility in diagnostic tests.

## Figures and Tables

**Table 2:** Assessment of the susceptibility to fluconazole of selected strains of *Candida* spp.

No.	Strain Number	Species	MIC [mg/L]	
			Clotrimazole	Fluconazole
1.	488	*C. albicans*	0.125	4 (I)
2.	1050	*C. albicans*	4	64 (R)
3.	1342	*C. albicans*	8	>64 (R)
4.	239	*C. glabrata*	2	4 (I)
5.	2445	*C. glabrata*	2	2 (I)
6.	984	*C. glabrata*	4	4 (I)
7.	136	*C. glabrata*	4	32 (R)
8.	2586	*C. glabrata*	4	64 (R)
9.	2922	*C. glabrata*	4	>64 (R)
10.	196	*C. glabrata*	8	64 (R)
11.	772	*C. glabrata*	8	64 (R)
12.	630	*C. guillermondii*	2	4 *
13.	678	*C. guillermondii*	1	4 *
14.	2933	*C. guillermondii*	1	4 *
15.	2408	*C. tropicalis*	1	4 (I)

Explanations to Table 2: Interpretation of the susceptibility to fluconazole according to EUCAST Breakpoint tables for the interpretation of MICs for antifungal agents, Version 10.0, valid from 4 February 2020 R, fluconazole-resistant strain I, susceptible, increased exposure to fluconazole * *Candida guilliermondii* is a species naturally having higher fluconazole MIC values than *Candida albicans*, and the epidemiological cut-off (ECOFFs) is 16 mg/L [4].

**Table 3:** Susceptibility of different Candida species to clotrimazole; data from the literature.

Reference	Method	Sample	Number of Strains Tested	Clotrimazole MIC mg/L				% of Non-WT
				Range	GM	MIC50	MIC90	
***C. albicans***								
Shi et al., 2020 [7]	CLSI, M27-A3	Vaginal	1272	0.015–32	0.05	-	0.25	ND
Rhichter et al., 2005 [5]	CLSI, M27A	Vaginal	420	0.008–0.125		0.03	0.06	0
Theill et al., 2016 [8]	CLSI, M27-A3	Vaginal	282	0.015–0.12	0.02		0.03	0
Zhou et al., 2016 [9]	CLSI, M27A	Vaginal	216	0.03–4	0.046	<0.03	0.25	ND
Diaz et al., 2016 [10]	CLSI, M27-A3	Vaginal	126	0.03–0.5		0.03	0.06	0
Choukri et al., 2014 [11]	CLSI, M27-A3	Vaginal	113	0.015–4	-	0.03	0.06	ND
Pelletier et al., 2000 [12]	CLSI, M27A	Oral (HIV+)	87	<0.06–8	-	<0.06	0.5	15 (17%)
Hacioglu et al., 2019 [13]	CLSI, M27-A3	Vaginal	84	0.01–2	-	0.007	0.25	ND
Nelson et al., 2013 [14]	CLSI, M27 A2	Vaginal	60	0.03–16	-	0.25	16	36.7%
Mesquida et al., 2021 [15]	EUCAST	Vaginal	72	0.004–0.25	0.006	0.008	0.06	0
Rezaei-Matehkolaei et al., 2016 [16]	microdilution home method	Vaginal	30	0.25–1	0.29	0.25	0.5	5 (16.6%)
Kiakojuri et al., 2021 [17]	CLSI	Auricular	16	1–16	2.484	1.5	16	ND
***C. dubliniensis***								
Theill et al., 2016 [8]	CLSI M27-A3	Vaginal	4	0.015–0.03				ND
Shi et al., 2020 [7]	CLSI, M27-A3	Vaginal	1	0.015				ND
***C. africana***								
Shi et al., 2020 [7]	CLSI, M27-A3	Vaginal	49	0.15–4	-	-	0.06	ND
Farahyar et al., 2020 [18]	CLSI, M27-S	Vaginal	3	0.06–16	-	-		1
Theill et al., 2016 [8]	CLSI M27-A3	Vaginal	1	0.03	-	-		ND
***C. glabrata***								
Shi et al., 2020 [7]	CLSI, M27-A3	Vaginal	267	0.015–16	0.2	-	1	ND
Costa et al., 2016 [6]	CLSI, M27-S4 /EUCAST	Varia	138	0.03–8	-	1	8	89 (64%)
Rhichter et al., 2005 [5]	CLSI, M27A	Vaginal	112	0.06–8	-	1	4	ND
Choukri et al., 2014 [11]	CLSI, M27-A3	Vaginal	54	0.25–8	-	2	4	ND
Lotfalikhani et al., 2018 [19]	CLSI, M27	Vaginal, blood	41	0.1–4	-	0.5	4	15 (30%)
Nelson et al., 2013 [14]	CLSI, M27 A2	Vaginal	28	0.03–16	-	0.125	0.25	10.7%
Diaz et al., 2016 [10]	CLSI, M27-A3	Vaginal	16	0.03–1		0.25	1	ND
Zhou et al., 2016 [9]	CLSI, M27A	Vaginal	13	0.03–2	0.2466	0.25	1	ND
Hacioglu et al., 2019 [13]	CLSI, M27-A3	Vaginal	9	0.25–2		0.5	2	ND
Rezaei-Matehkolaei et al., 2016 [16]	microdilution home method	Vaginal	3	0.25		0.06	0.125	0
***C. nivariensis***								
Shi et al., 2020 [7]	CLSI, M27-A3	Vaginal	9	0.03–0.5				0
***C. bracariensis***								
Shi et al., 2020 [7]	CLSI, M27-A3	Vaginal	2	0.03–0.5				0
***C. krusei***								
Shi et al., 2020 [7]	CLSI, M27-A3	Vaginal	54	0.015–0.5	0.08	-	0.5	0%
Singh et al., 2002 [20]	CLSI, M27-A	Vaginal	26	0.03–0.5	-	0.125	0.25	0%
Rhichter et al., 2005 [7]	CLSI, M27A	Vaginal	12	0.125–1	-	0.25	0.5	1 (3.5%)
Choukri et al., 2014 [11]	CLSI, M27-A3	Vaginal	11	0.25–0.5		0.5	0.5	0%
Nelson et al., 2013 [14]	CLSI, M27-A2	Vaginal	2	1–4				2 (100%)
Hacioglu et al., 2019 [13]	CLSI, M27-A3	Vaginal	1	0.5				0%
***C. parapsilosis***								
Shi et al., 2020 [6]	CLSI, M27-A3	Vaginal	76	0.015–1	0.04	-	0.06	ND
Rhichter et al., 2005 [7]	CLSI, M27A	Vaginal	30	0.03–0.5	-	0.06	0.25	0%
Choukri et al., 2014 [11]	CLSI, M27-A3	Vaginal	11	0.03–0.5		0.12	0.12	0%
Kiakojuri et al., 2021 [17]	CLSI	auricular	12	1–>16	3.364	2	16	12 (100%)
Diaz et al., 2016 [10]	CLSI, M27-A3	Vaginal	2	0.03				0%
Nelson et al., 2013 [14]	CLSI, M27 A2	Vaginal	1	0.125				0%
***C. metapsilosis***								
Shi et al., 2020 [6]	CLSI, M27-A3	Vaginal	20	0.03–0.25	0.04	-	0.06	0%
***C. orthopsilosis***								
Kiakojuri et al., 2021 [17]	CLSI	Auricular	18	0.25–>16	1.782	1	16	
Shi et al., 2020 [6]	CLSI, M27-A3	Vaginal	6	0.03–1			1	
***C. tropicalis***								
Shi et al., 2020 [6]	CLSI, M27-A3	Vaginal	61	0.015–0.25	0.05	-	0.05	0
Choukri et al., 2014 [11]	CLSI, M27-A3	Vaginal	11	0.12–0.25	-	0.12	0.25	0
Rhichter et al., 2005 [7]	CLSI, M27A	Vaginal	8	0.03–0.25	-			0
Nelson et al., 2013 [14]	CLSI, M27 A2	Vaginal	3	0.125–2				1 (33.3%)
Hacioglu et al., 2019 [13]	CLSI, M27-A3	Vaginal	2	0.007–0.015				0
Kiakojuri et al., 2021 [17]	CLSI	Auricular	1	4				1 (100%)
Diaz et al., 2016 [10]	CLSI, M27-A3	Vaginal	1	1				1(100%)
***C. kefyr***								
Hacioglu et al., 2019 [13]	CLSI, M27-A3	Vaginal	2	0.007–0.5				
Rezaei-Matehkolaei et al., 2016 [16]	microdilution home method	Vaginal	1	0.25				
***C. auris***								
Kiakojuri et al., 2021 [17]	CLSI	auricular	1	8				
***C. lusitniae***								
Rhichter et al., 2005 [7]	CLSI, M27A	Vaginal	1	0.06				
Hacioglu et al., 2019 [13]	CLSI, M27-A3	Vaginal	1	0.5				

Abbreviations: ND, no data; non-WT, non-wild-type phenotype.

## Data Availability

Data are contained within the article or supplementary material.

## Supplementary Materials

The following are available online at https://www.mdpi.com/article/10.3390/pathogens10091142/s1, Table S1: List of tested strains, their origin, and susceptibility results.
